# Virulence-Associated Characteristics of Serotype 14 and Serogroup 9 *Streptococcus pneumoniae* Clones Circulating in Brazil: Association of Penicillin Non-susceptibility With Transparent Colony Phenotype Variants

**DOI:** 10.3389/fmicb.2020.02009

**Published:** 2020-08-26

**Authors:** Tatiana C. A. Pinto, Natália S. Costa, Sandrine E. C. M. Pina, Aline R. V. Souza, Laura M. A. Oliveira, Camille A. B. Moura, Fabíola C. O. Kegele, Vânia L. C. Merquior, Ana Caroline N. Botelho, José M. Peralta, Lúcia M. Teixeira

**Affiliations:** ^1^Instituto de Microbiologia Paulo de Góes, Universidade Federal do Rio de Janeiro, Rio de Janeiro, Brazil; ^2^Instituto Fernandes Figueira, Fundação Oswaldo Cruz, Rio de Janeiro, Brazil; ^3^Departamento de Microbiologia, Imunologia e Parasitologia, Universidade do Estado do Rio de Janeiro, Rio de Janeiro, Brazil

**Keywords:** *Streptococcus pneumoniae*, pneumococcal surface protein A, pilus type 1, colony phase variation, penicillin-non-susceptible pneumococci

## Abstract

*Streptococcus pneumoniae* remains a major agent of invasive diseases, especially in children and the elderly. The presence of pneumococcal capsule, pneumococcal surface protein A (PspA), and pilus type 1 (PI-1) and the ability of colony phase variation are assumed to play important roles in the virulence potential of this microorganism. Differences in the capsular polysaccharide allow the characterization of more than 90 pneumococcal serotypes; among them, serotype 14 and serogroup 9 stand out due to their prevalence in the pre- pneumococcal conjugate vaccine era and frequent association with penicillin non-susceptibility. Here we investigated the distribution of PI-1 and *psp*A genes and colony phase variants among 315 *S. pneumoniae* isolates belonging to serotype 14 and serogroup 9, recovered over 20 years in Brazil, and correlated these characteristics with penicillin susceptibility and genotype as determined by multilocus sequence typing. All strains were shown to carry *psp*A genes, with those of family 2 (*psp*A2) being the most common, and nearly half of the strains harbored P1-1 genes. The *psp*A gene family and the presence of PI-1 genes were conserved features among strains belonging to a given clone. A trend for increasing the occurrence of *psp*A2 and PI-1 genes over the period of investigation was observed, and it coincided with the dissemination of CC156 (Spain^9*V*^-3) clone in Brazil, suggesting a role for these virulence attributes in the establishment and the persistence of this successful clone. Opaque variant was the colony phenotype most frequently observed, regardless of clonal type. On the other hand, the transparent variant was more commonly associated with penicillin-non-susceptible pneumococci and with strains presenting evidence of recombination events involving the genes coding for polysaccharide capsule and PspA, suggesting that pneumococcal transparent variants may present a higher ability to acquire exogenous DNA. The results bring to light new information about the virulence potentials of serotype 14 and serogroup 9 *S. pneumoniae* isolates representing the major clones that have been associated with the emergence and the dissemination of antimicrobial resistance in our setting since the late 1980s.

## Introduction

*Streptococcus pneumoniae* is a leading cause of invasive infections and can also asymptomatically colonize the upper respiratory tract of variable numbers of individuals (Weiser et al., 2018). The polysaccharide capsule is considered as the major virulence factor of this species, and its antigenic diversity allows the characterization of pneumococcal isolates into more than 90 serotypes (Geno et al., 2015). Additionally, several other virulence factors contribute to the pathogenic potential of this microorganism, including the pneumococcal surface protein A (PspA), which reduces opsonization and clearance of bacteria by the host immune system (Kadioglu et al., 2008), and the pilus type 1 (PI-1), which is believed to mediate the attachment of pneumococci to human epithelial cells and the extracellular matrix (Nelson et al., 2007; Hilleringmann et al., 2008; Iovino et al., 2020). PspA is known to occur in virtually all pneumococcal strains and exhibits polymorphic regions, being classified in three families and six clades (Hollingshead et al., 2000; Croney et al., 2012). The occurrence of PI-1, in turn, seems to be restricted to certain pneumococcal clones (Sjöström et al., 2007; Aguiar et al., 2008; Imai et al., 2011; Selva et al., 2012; Metcalf et al., 2016), but its distribution among pneumococcal isolates circulating in different geographic areas, including Brazil, has not been extensively investigated yet. The virulence of *S. pneumoniae* is also associated with phase variation, a phenomenon characterized by the expression of two colony phenotypes, opaque and transparent. These variants may differ from each other in multiple virulence-associated characteristics (Li et al., 2016; Li and Zhang, 2019). In addition, PI-1 was shown to have an on/off regulation, which has been associated with phase variation (Basset et al., 2011; Danne et al., 2014).

The efforts led by the Pneumococcal Molecular Epidemiology Network (PMEN) have helped in tracking successful pneumococcal clones disseminated worldwide, which are usually associated with antimicrobial resistance^1^. In Brazil, we have shown that four major clonal complexes (CC) were responsible for the emergence and the dissemination of antimicrobial resistance among *S. pneumoniae* strains of serotypes 14 and 9 circulating in the country since the late 1980s (Pinto et al., 2016). Among them, three are related to PMEN clones, including CC156 (related to Spain^9*V*^-3), CC66 (related to Tennessee^14^-18), and CC15 (related to England^14^-9), while the fourth, namely CC5401, is characterized as a regional clone. Despite the importance of such CCs in the dissemination of antimicrobial resistance, little is known about other characteristics that could potentially influence their pathogenic potential. Therefore, in the present study, we have evaluated the distribution of three virulence-associated characteristics (presence of *psp*A and PI-1 coding genes, as well as colony phase variation phenotypes), among *S. pneumoniae* isolates belonging to major clones of serotype 14 or serogroup 9 circulating in Brazil, which were recognized to be responsible for the emergence and the expansion of penicillin non-susceptibility in our setting.

## Materials and Methods

### Bacterial Strains

A total of 315 *S. pneumoniae* isolates were investigated, encompassing 216 of serotype 14 and 99 of serogroup 9. All the isolates were previously characterized regarding capsular type, antimicrobial susceptibility profile, and multilocus sequence typing (Pinto et al., 2016). A number of them (89 isolates) were also previously characterized by multiple locus variable-number tandem repeat (VNTR) analysis (MLVA; Costa et al., 2016).

In addition, reference strains belonging to five worldwide disseminated pneumococcal lineages (Spain^9*v*^-3 ST156, England^14^-9 ST9, Tennessee^14^-18 ST67, Netherlands^14^-35 ST124, and Netherlands^15*B*^-37 ST199), characterized by the PMEN^1^ were included. Such lineages were previously shown to be associated with certain isolates included in the present study (Pinto et al., 2016).

### *psp*A Typing

The determination of *psp*A gene type was carried out by PCR as previously described (Pimenta et al., 2006), with a modification in the MgCl_2_ concentration (used at 3 mM in all PCR mixtures in the present study). Bacterial DNAs were obtained by using the Chelex^®^ 100 resin (Bio-Rad Laboratories, United States) as previously described (Pinto et al., 2013).

*psp*A typing (*psp*A1, *psp*A2, and *psp*A3) was performed for all 315 isolates included in the study by using specific primers for family 1 (LSM12 and SKH63, Swiatlo et al., 1997; Vela-Coral et al., 2001), family 2 (LSM12 and SKH52, Swiatlo et al., 1997; Vela-Coral et al., 2001), and family 3 (SKH41 and SKH42; Hollingshead et al., 2006). The isolates that were not initially amplified using an annealing temperature of 62°C were retested under the same cycling conditions but using annealing temperatures of 58°C and, subsequently, 55°C. The *psp*A clade (1–6) was determined for a subset of 34 strains selected to represent the most important clones previously detected (Pinto et al., 2016) and required an additional sequencing step after the PCR amplification of the clade-defining region (CDR) using primers LSM12 and SKH2 (Swiatlo et al., 1997; Hollingshead et al., 2000). The amplification products were purified using ExoSAP-IT (Affymetrix-USB, United States) according to the manufacturer’s instructions and were run on an ABI 3130 Genetic Analyzer (Applied Biosystems, United States). The sequences were edited and aligned with BioEdit v7.0.9.0 (Hall, 1999).

The *psp*A gene families and clades were also determined for the reference strains of the five PMEN clones included in the study. *S. pneumoniae* reference strains were included as controls for *psp*A clade 1 (strain BG9739), clade 2 (strain EF10197), clade 3 (strain AC122), clade 4 (strain BG7561), clade 5 (ATCC 6303), and clade 6 (strain BG6380).

The recombination events among *psp*A genes of 15 *S. pneumoniae* strains, selected to represent different sequence types (ST) within the clonal complex CC156, were identified *in silico* using the recombination detection program (RDP) package, which incorporates the RDP, GENECONV, Maxchi, Chimera, 3Seq, Bootscan, and SiSscan programs (Martin et al., 2015), to predict the recombination signals from aligned DNA sequences. The recombination events were scored as significant only if at least three out of seven individual programs in the package identified the events with *p* < 0.05.

### Detection of PI-1 Coding Genes

The presence of PI-1 coding genes was evaluated among all 315 pneumococcal strains included in the study and also among the reference strains of five PMEN clones. Bacterial DNAs were obtained by using the Chelex^®^ 100 resin (Pinto et al., 2013), and the presence of genes associated with PI-1 production was evaluated using the primer set described by Moschioni et al. (2008). Controls for the presence (pneumococcal reference strain TIGR4) and absence (pneumococcal reference strain R6) of PI-1 genes were included in each set of reactions.

### Microscopic Observation and Quantification of Pneumococcal Colony Phase Variation

A microscopic examination of pneumococcal colony phase variation was carried out for the 235 strains as previously described (Weiser et al., 1994). Briefly, bacteria were grown on 5% sheep blood agar plates (Plast Labor, Brazil) for 18–24 h at 37°C under 5%-CO_2_ atmosphere. A single colony of each strain was inoculated onto a plate containing Todd-Hewitt agar (BBL, United States), supplemented with 0.5% yeast extract (Difco, United States), and incubated for 18–24 h at 37°C in 5% CO_2_. Subsequently, the colonies on each plate were submitted to microscopic examination and were assigned to either opaque or transparent colony morphology by using a stereoscopic microscope equipped with a × 40 magnifying glass (TecNival, Brazil).

### Statistical Analysis

The evaluation of the distribution of colony phase variants among penicillin-susceptible and penicillin-non-susceptible strains and also among ST156 and ST162 isolates was carried out by applying the one-way analysis of variance (ANOVA) test. The distribution of colony variants, *psp*A types, and PI-1 coding genes over the years was investigated by linear regression analysis. GraphPad Prism software v5.0 was used to perform all the statistical analysis and *p* < 0.05 were considered as significant.

## Results and Discussion

The characteristics of the 315 *S. pneumoniae* isolates analyzed in the present study are included in Supplementary Table S1.

All 315 isolates harbored *psp*A genes, demonstrating the wide occurrence of these genes among pneumococcal isolates as previously suggested (McDaniel et al., 1998; Croney et al., 2012; Blumental et al., 2015; Kawaguchiya et al., 2018; Knupp-Pereira et al., 2019). A total of 30.8% (97 isolates) harbored *psp*A1 genes, while 69.2% (218 isolates) had *psp*A2 genes. The *psp*A3 genes were not detected. These observations reinforce the concept that about 98% of the pneumococcal strains circulating worldwide are estimated to belong to families 1 or 2, while the occurrence of family 3 is rare (Ochs et al., 2008; Blumental et al., 2015; Kawaguchiya et al., 2018; Knupp-Pereira et al., 2019). The isolates possessing the *psp*A1 gene that were submitted to CDR sequencing (10 in total) were included in clade 1, while the *psp*A2 isolates analyzed (24 in total) belonged to clade 3. Types of *psp*A genes were highly conserved among strains within each clone (Table 1). The association of CC156 with *psp*A2 and clade 3 observed in this study has already been detected among isolates from different countries (Melin et al., 2008; Rolo et al., 2009; Sadowy et al., 2010), as well as the association of CC15 with *psp*A1 and clade 1 (Ito et al., 2007; Melin et al., 2008; Rolo et al., 2009), showing that the PMEN-related clones identified in Brazil share characteristics with those circulating internationally.

**TABLE 1:** Characteristics of the clonal complexes (CC) and singleton sequence types (ST) of the 315 *Streptococcus pneumoniae* isolates included in the present study.

**CC (number of isolates)**		**Serotypes**	**% PNSP**	***psp*A type (%)**	**PI-1 genes (%)**	**PMEN clone-associated**
CC156 (149)	Subcluster ST162 (40)	9V, 9A	5%	*psp*A2 clade 3 (97.5%)	Present (100%)	Spain^9V^-3 ST156
	Subcluster ST156 (109)	14, 9V, 9A	99.1%	*psp*A2 clade 3 (96.3%)	Present (98.2%)	
CC66 (67)		14, 9N, 9L	47.8%	*psp*A2 clade 3 (94%)	Absent (98.5%)	Tennessee^14^-18 ST66
CC15 (59)		14	8.5%	*psp*A1 clade 1 (96.1%)	Absent (100%)	England^14^-9 ST9
CC5401 (22)		14	22.7%	*psp*A1 clade 1 (100%)	Absent (100%)	NA
CC124 (5)		14	None	*psp*A2 clade 4 (60%)	Absent (80%)	Netherlands^14^-35 ST124
CC7041 (2)		14	None	*psp*A1 (100%)	Present (100%)	NA
CC796 (2)	ST796 (1)	14	None	*psp*A1 (100%)	Absent (100%)	NA
	ST7113 (1)	14	100%	*psp*A2 (100%)	Present (100%)	
ST280 (4)		9V, 9A	None	*psp*A1 clade 1 (100%)	Absent (100%)	NA
ST199 (3)		14	33.3%	*psp*A2 clade 4 (100%)	Absent (100%)	Netherlands^15*B*^-37 ST199
ST7047 (1)		14	None	*psp*A2 (100%)	Present (100%)	NA
ST7088 (1)		14	None	*psp*A2 (100%)	Absent (100%)	NA

**PNSP, penicillin-non-susceptible pneumococci; pspA1, pspA genes of family 1; pspA2, pspA genes of family 2; PI-1, pilus type 1; PMEN, Pneumococcal Molecular Epidemiology Network; NA, not applicable.**

Around 50% of the isolates (total of 154) had PI-1 genes, and the presence of these determinants varied according to the clone (Table 1). CC156, CC7041, ST7113, and ST7047 were the only genotypes associated with PI-1. Accordingly, previous studies have revealed that PI-1 is harbored by certain pneumococcal clones, especially among the internationally disseminated ST156 (Sjöström et al., 2007; Aguiar et al., 2008; Imai et al., 2011; Selva et al., 2012; Horácio et al., 2016). In turn, CC7041, ST7113, and ST7047 are genotypes described only in Brazil up to date (accessed on April 4th 2020)^2^, and, thus, their association with PI-1 is being described here for the first time.

Among the five PMEN clones investigated in the present study, all had *psp*A genes and two harbored PI-1 coding genes, including Spain^9*V*^-3 ST156 and Netherlands^15*B*^-37 ST199 (Table 2). In general, the reference strains of PMEN clones presented profiles of *psp*A type and PI-1 genes identical to those of clinical isolates associated with these clones (Table 1). The only exception regarding the presence of PI-1 genes within Netherlands^15*B*^-37 ST199 isolates is that, while the respective reference strain harbored PI-1 genes (Table 2), three clinical isolates belonging to ST199 evaluated in the present study did not (Table 1). This finding is in contrast with a previous report indicating the association of PI-1 with clinical isolates belonging to CC199 from Italy (Del Grosso et al., 2013). However, ST199 isolates usually belong to serogroups 15 or 19^2^, and the ones included in the present study were of serotype 14, representing the first serotype 14 variants of this clone to be reported (Pinto et al., 2016). We have also previously shown that these serotype 14 ST199 variants might have been generated by capsular switching events since they were genetically more closely related to strains belonging to serotype 15B than to other isolates of serotype 14 (Costa et al., 2016). Previous studies have indicated that capsular switching events in pneumococci can lead to other simultaneous recombination events spanning a genome region of approximately 750 kb, which includes the capsular locus and the pilus type 1 operon (Sjöström et al., 2007; Croucher et al., 2014; Metcalf et al., 2016); thus, in these serotype 14 ST199 variants, PI-1 coding genes could have been lost during recombination events in the capsular locus that led to serotype switching.

**TABLE 2:** Profiles of *psp*A gene types and pilus type 1 coding genes of five reference strains representative of Pneumococcal Molecular Epidemiology Network (PMEN) *Streptococcus pneumoniae* clones included in the present study.

**PMEN clone (number of reference strain)**	***psp*A type**	**PI-1**
Spain^9*V*^-3 ST156 (ATCC 700671)	*psp*A2 clade 3	Present
England^14^-9 ST9 (ATCC 700676)	*psp*A1 clade 1	Absent
Tennessee^14^-18 ST67 (ATCC BAA-340)	*psp*A2 clade 3	Absent
Netherlands^14^-35 ST124 (ATCC BAA-1661)	*psp*A2 clade 4	Absent
Netherlands^15*B*^-37 ST199 (ATCC BAA-1663)	*psp*A2 clade 4	Present

**pspA1, pspA genes of family 1; pspA2, pspA genes of family 2; PI-1, pilus type 1.**

Not all 315 pneumococcal isolates were tested for colony phenotype. Although this represents a limitation of the study, it is expected that the results obtained from the subset of 235 (almost 75% of the total) isolates are highly representative of the total sampling as they were selected to encompass the different variants included in the study. The majority of the 235 pneumococcal isolates submitted to the determination of colony phenotype (128 isolates, 54.5%) consisted of exclusively opaque variants, while 19.6% (46 isolates) were exclusively transparent and 25.9% (61 isolates) presented mixed phenotypes comprising both opaque and transparent variants. The opaque phenotype was the most common overall, corroborating previous observations (Arai et al., 2011). The distribution of opaque and transparent variants did not correlate with *psp*A types or presence of PI-1 coding genes. The four major clonal complexes included in the study (CC156, CC66, CC15, and CC5401) likewise presented very similar distributions of both opaque and transparent variants, with a slightly higher frequency of transparent variants within CC5401 (Table 3).

**TABLE 3:** Distribution of colony phase variants among the four major clonal complexes (CCs) of serotypes 9 and 14 *Streptococcus pneumoniae* isolates included in this study.

**Clonal complex**	**Colony phase variants**		
	**Exclusively opaque (%)**	**Mixed (opaque + transparent) (%)**	**Exclusively transparent (%)**
CC156 (*n* = 103)	55	26	19
ST156 subcluster (*n* = 68)	48	28	24
ST162 subcluster (*n* = 35)	68	23	9
CC66 (*n* = 49)	49	31	20
CC15 (*n* = 52)	58	25	17
CC5401 (*n* = 15)	54	13	33

On the other hand, the distribution of opaque and transparent variants between penicillin-susceptible (PSP) and penicillin-non-susceptible (PNSP) isolates was markedly different, with a significantly higher frequency of transparent variants and a lower occurrence of opaque variants within PNSP isolates (Table 4; *p* = 0.0126). Similarly, PNSP occurrence among isolates showing the transparent phenotype (56.5%) was much more frequent than among those presenting the opaque phenotype (37.5%). PNSP in this study comprised all isolates that were shown to present penicillin minimal inhibitory concentrations (MIC) ≥0.12 μg/ml (Pinto et al., 2016).

**TABLE 4:** Distribution of colony phase variants among serotypes 9 and 14 *Streptococcus pneumoniae* isolates according to the penicillin susceptibility profile.

**Penicillin susceptibility**	**Colony phase variants**		
	**Exclusively opaque (%)**	**Mixed (opaque + transparent) (%)**	**Exclusively transparent (%)**
PNSP (*n* = 100)	48	26	26
PSP (*n* = 135)	59	26	15

**PSP, penicillin-susceptible pneumococci; PNSP, penicillin-non-susceptible pneumococci.**

We have shown earlier that the emergence of PNSP and the increment on penicillin MICs among pneumococcal isolates circulating in our setting were mainly due to the introduction of ST156 in the mid-1990s (Pinto et al., 2016). Additionally, it has been suggested that ST156 was derived from a penicillin-susceptible ancestor, the genotype ST162, which is a single-locus variant (SLV) of ST156 (Sjöström et al., 2007). Therefore, although ST156 and ST162, as well as their SLVs, are components of CC156, they can be analyzed as two different subclusters, especially with regard to penicillin susceptibility (Table 1). Interestingly, a significant difference (*p* = 0.0151) in the distribution of colony phenotypes was also seen between the two subclusters included in CC156 (Table 3). The isolates belonging to subcluster ST156, associated with penicillin non-susceptibility, presented higher and lower frequencies of transparent and opaque variants, respectively, when compared to subcluster ST162. Such observations may indicate a correlation between the transparent phenotype and penicillin non-susceptibility among the pneumococcal isolates investigated, but further studies are required to better elucidate this question.

The opaque phenotype was the most common overall and also within each one of all the clones investigated in this study, except for ST199 isolates, which we have shown to be a probable result of capsular switching events (Costa et al., 2016). Among these three serotype 14 ST199 variants, two were exclusively composed of transparent variants, while the third was composed of a mixed phenotype, indicating that the genotype in which capsular recombination events were assumed to occur was also the only genotype where the transparent phenotype was more frequent.

The association of the transparent phenotype with higher rates of recombination in *psp*A genes was also detected in this study. Among the 15 pneumococcal isolates submitted to *in silico* evaluation of recombination events in *psp*A genes, five had significant signals of recombination; all of them belonged to subcluster ST156 and three were shown to be exclusively composed of transparent variants, while the other two comprised mixed phenotypes. On the other hand, none of the isolates presenting exclusively opaque variants had significant signals of recombination in *psp*A genes (Table 5).

**TABLE 5:** Characteristics of the 15 *Streptococcus pneumoniae* isolates belonging to different subclusters of clonal complex 156 (CC156) submitted to *in silico* analysis of recombination events in *psp*A genes by using the recombination detection program software.

**Strain**	**Subcluster**	**Colony phenotype**	**Penicillin susceptibility**	**Serotype**	**Recombination signals**
Sp904	ST162	Opaque	PSP	9V	Absent
Sp911	ST156	Opaque	PNSP	14	Absent
Sp979	ST156	Transparent	PNSP	14	Present
Sp985	ST156	Transparent	PNSP	14	Present
Sp1001	ST156	Opaque	PNSP	14	Absent
Sp1033	ST156	Opaque	PNSP	14	Absent
Sp1040	ST156	Opaque	PNSP	14	Absent
Sp1316	ST156	Opaque	PNSP	14	Absent
Sp1353	ST156	Mixed	PSP	9A	Present
Sp1428	ST162	Opaque	PSP	9V	Absent
Sp1484	ST156	Transparent	PNSP	14	Present
Sp1655	ST162	Opaque	PSP	9V	Absent
Sp1916	ST162	Transparent	PNSP	9V	Absent
Sp2152	ST156	Mixed	PNSP	14	Absent
Sp2196	ST156	Mixed	PNSP	14	Present

**PSP, penicillin-susceptible pneumococci; PNSP, penicillin-non-susceptible pneumococci.**

Although phase variation in pneumococci has been investigated over the past decades, the molecular mechanisms involved in this phenomenon are still unclear (Weiser et al., 1994; Manso et al., 2014; Li et al., 2016; Li and Zhang, 2019). Earlier studies have shown that it appears to be independent of *in vitro* growth conditions including pH, temperature, and osmolarity (Kim et al., 1999). In addition, genetic analyses have demonstrated that BOX elements (repeat sequences in the pneumococcal genome) can have a role in the frequency of phase variation (Saluja and Weiser, 1995; Li et al., 2016). Many of the 315 pneumococcal isolates included in this study were previously analyzed by MLVA (Costa et al., 2016), a methodology that targets BOX elements. This previous study showed interesting differences in the number of repeat sequences in certain BOX elements between ST156 and ST162 isolates. In the BOX element represented by *locus* Spneu40, for example, two or three repeats were found in ST162 isolates, while a number of eight repeats were observed in ST156 isolates (Costa et al., 2016).

**FIGURE 1:**
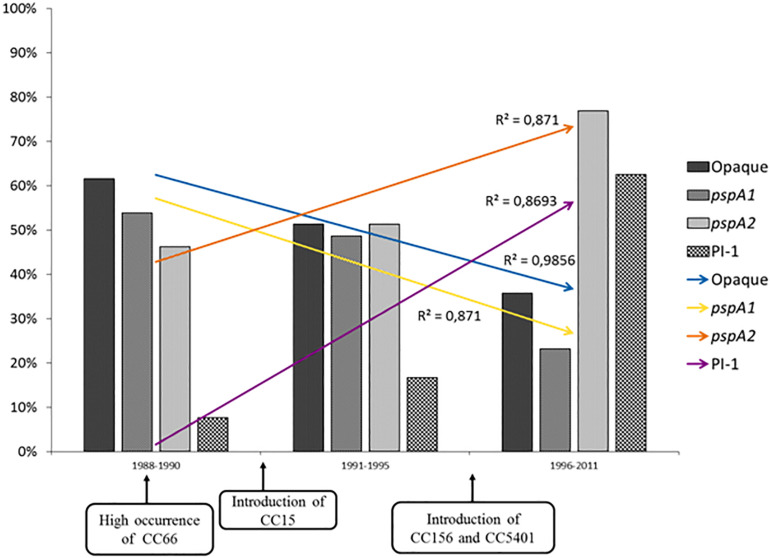
Distribution of opaque variants, *psp*A gene types, and pilus type 1 (PI-1) coding genes among serotypes 9 and 14 *Streptococcus pneumoniae* isolates investigated in this study over the years. Opaque, isolates with opaque phenotype; *psp*A1, *psp*A genes of family 1; *psp*A2, *psp*A genes of family 2.

In the present investigation, an increasing trend for the occurrence of isolates harboring *psp*A2 and PI-1 genes was observed over the years, in parallel with a decreasing tendency of isolates showing *psp*A1 genes and the opaque phenotype (Figure 1). These trends might actually reflect the natural fluctuation of clonal complexes over time as it was previously shown that ST156, which is associated with PI-1 and *psp*A2 genes and a higher frequency of transparent variants, has been increasing steadily in occurrence since the mid-1990s (Pinto et al., 2016). However, changes in the occurrence of PI-1 and PspA families have been observed in the United States after the implementation of the seven-valent pneumococcal conjugate vaccine (Regev-Yochay et al., 2010; Croney et al., 2012). Since this study only included isolates recovered before the introduction of the first pneumococcal conjugate vaccine (10-valent PCV) offered to the general population by the Brazilian public health service, the continuous tracking of these characteristics among isolates obtained in more recent years is required and may reveal the impact of PCV10 in the distribution of virulence-associated characteristics among pneumococci.

This study highlights data on the virulence potential of serotype 14 and serogroup 9 *S. pneumoniae* isolates representing the major CCs that have been responsible for the emergence and the dissemination of antimicrobial resistance in our setting since the late 1980s. Particularly, the results indicate that important recombination events involving the genes coding for penicillin-binding proteins (leading to penicillin non-susceptibility), capsule polysaccharide (leading to serotype switching), and PspA are more associated with the transparent variants of pneumococci, which may present an improved ability to acquire exogenous DNA. The evaluation of such virulence-associated characteristics can help to better understand the evolution and the adaptation of pneumococcal clones over the years and, consequently, may be helpful in designing new strategies to prevent pneumococcal diseases.

## Data Availability Statement

All datasets generated for this study are included in the article/Supplementary Material.

## Author Contributions

TP, VM, JP, and LT designed the study. CM and FK performed the microscopic analysis of colony variants. NC, AS, and AB performed the experiments for pilus gene detection. SP, LO, and AB performed the experiments for *psp*A typing. TP performed *in silico* and statistical analysis. TP, VM, JP, and LT wrote the manuscript. All authors contributed to the article and approved the submitted version.

## Conflict of Interest

The authors declare that the research was conducted in the absence of any commercial or financial relationships that could be construed as a potential conflict of interest.

## Footnotes

1 www.pneumogen.net/pmen/

2 http://pubmlst.org/spneumoniae/

## Supplementary Material

TABLE S1 Characteristics of the 315 *Streptococcus pneumoniae* isolates included in the present study. ^*a*^ND, not determined, ^*b*^Penicillin minimum inhibitory concentration, ^*c*^Sequence type.

## References

[B1] Aguiar S. I. Serrano I. Pinto F. R. Melo-Cristino J. Ramirez M. (2008). The presence of the pilus locus is a clonal property among pneumococcal invasive isolates. *BMC Microbiol.* 8:41. 10.1186/1471-2180-8-41 18307767 PMC2270847

[B2] Arai J. Hotomi M. Hollingshead S. K. Ueno Y. Briles D. E. Yamanaka N. (2011). *Streptococcus pneumoniae* isolates from middle ear fluid and nasopharynx of children with acute otitis media exhibit phase variation. *J. Clin. Microbiol.* 49 1646–1649. 10.1128/jcm.01990-10 21346044 PMC3122802

[B3] Basset A. Turner K. H. Boush E. Sayeed S. Dove S. L. Malley R. (2011). Expression of the type 1 pneumococcal pilus is bistable and negatively regulated by the structural component RrgA. *Infect. Immun.* 79 2974–2983. 10.1128/iai.05117-11 21576325 PMC3147576

[B4] Blumental S. Granger-Farbos A. Moïsi J. C. Soullié B. Leroy P. Njanpop-Lafourcade B. M. (2015). Virulence factors of *Streptococcus pneumoniae*. Comparison between African and French invasive isolates and implication for future vaccines. *PLoS One* 10:e0133885. 10.1371/journal.pone.0133885 26214695 PMC4516325

[B5] Costa N. S. Pinto T. C. Merquior V. L. Castro L. F. da Rocha F. S. Morais J. M. (2016). MLVA Typing of *Streptococcus pneumoniae* isolates with emphasis on serotypes 14, 9N and 9V: comparison of previously described panels and proposal of a novel 7 VNTR loci-based simplified scheme. *PLoS One* 11:e0158651 10.1371/journal.pone.00158651 PMC4938579 27391462

[B6] Croney C. M. Coats M. T. Nahm M. H. Briles D. E. Crain M. J. (2012). PspA family distribution, unlike capsular serotype, remains unaltered following introduction of the heptavalent pneumococcal conjugate vaccine. *Clin. Vaccine Immunol.* 19 891–896. 10.1128/cvi.05671-11 22539473 PMC3370451

[B7] Croucher N. J. Chewapreecha C. Hanage W. P. Harris S. R. McGee L. van der Linden M. (2014). Evidence for soft selective sweeps in the evolution of pneumococcal multidrug resistance and vaccine escape. *Genome Biol. Evol.* 6 1589–1602. 10.1093/gbe/evu120 24916661 PMC4122920

[B8] Danne C. Dubrac S. Trieu-Cuot P. Dramsi S. (2014). Single cell stochastic regulation of pilus phase variation by an attenuation-like mechanism. *PLoS Pathog.* 10:e1003860 10.1371/journal.pone.1003860 PMC3894217 24453966

[B9] Del Grosso M. Camilli R. D’Ambrosio F. Petrucci G. Melchiorre S. Moschioni M. (2013). Increase of pneumococcal serotype 19A in Italy is due to expansion of the piliated clone ST416/CC199. *J. Med. Microbiol.* 62 1220–1225. 10.1099/jmm.0.061242-0 23722433

[B10] Geno K. A. Gilbert G. L. Song J. Y. Skovsted I. C. Klugman K. P. Jones C. (2015). Pneumococcal capsules and their types: past, present, and future. *Clin. Microbiol. Rev.* 28 871–899. 10.1128/cmr.00024-15 26085553 PMC4475641

[B11] Hall T. (1999). BioEdit: a user-friendly biological sequence alignment editor and analysis program for Windows 95/98/NT. *Nuclear Acids Symp. Series* 41 95–98.

[B12] Hilleringmann M. Giusti F. Baudner B. C. Masignani V. Covacci A. Rappuoli R. (2008). Pneumococcal pili are composed of protofilaments exposing adhesive clusters of RrgA. *PLoS Pathog.* 4:e1000026 10.1371/journal.pone.1000026 PMC2265430 18369475

[B13] Hollingshead S. K. Baril L. Ferro S. King J. Coan P. Briles D. E. (2006). Pneumococcal surface protein A (PspA) family distribution among clinical isolates from adults over 50 years of age collected in seven countries. *J. Med. Microbiol.* 55 215–221. 10.1099/jmm.0.46268-0 16434715

[B14] Hollingshead S. K. Becker R. Briles D. E. (2000). Diversity of PspA: mosaic genes and evidence for past recombination in *Streptococcus pneumoniae*. *Infect. Immun.* 68 5889–5900. 10.1128/iai.68.10.5889-5900.2000 10992499 PMC101551

[B15] Horácio A. N. Silva-Costa C. Diamantino-Miranda J. Lopes J. P. Ramirez M. Melo-Cristino J. (2016). Population structure of *Streptococcus pneumoniae* causing invasive disease in adults in Portugal before PCV13 availability for adults: 2008-2011. *PLoS One* 11:e0153602. 10.1371/journal.pone.0153602 27168156 PMC4864403

[B16] Imai S. Ito Y. Ishida T. Hirai T. Ito I. Yoshimura K. (2011). Distribution and clonal relationship of cell surface virulence genes among *Streptococcus pneumoniae* isolates in Japan. *Clin. Microbiol. Infect.* 17 1409–1414. 10.1111/j.1469-0691.2010.03446.x 21143699

[B17] Iovino F. Nannapaneni P. Henriques-Normark B. Normark S. (2020). The impact of the ancillary pilus-1 protein RrgA of *Streptococcus pneumoniae* on colonization and disease. *Mol. Microbiol.* 113 650–658. 10.1111/mmi.14451 32185835

[B18] Ito Y. Osawa M. Isozumi R. Imai S. Ito I. Hirai T. (2007). Pneumococcal surface protein A family types of *Streptococcus pneumoniae* from community-acquired pneumonia patients in Japan. *Eur. J. Clin. Microbiol. Infect. Dis.* 26 739–742. 10.1007/s10096-007-0364-7 17665229

[B19] Kadioglu A. Weiser J. N. Paton J. C. Andrew P. W. (2008). The role of *Streptococcus pneumoniae* virulence factors in host respiratory colonization and disease. *Nat. Rev. Microbiol.* 6 288–301. 10.1038/nrmicro1871 18340341

[B20] Kawaguchiya M. Urushibara N. Aung M. S. Morimoto S. Ito M. Kudo K. (2018). Genetic diversity of pneumococcal surface protein A (PspA) in paediatric isolates of non-conjugate vaccine serotypes in Japan. *J. Med. Microbiol.* 67 1130–1138. 10.1099/jmm.0.000775 29927374

[B21] Kim J. O. Romero-Steiner S. Sørensen U. B. Blom J. Carvalho M. Barnard S. (1999). Relationship between cell surface carbohydrates and intrastrain variation on opsonophagocytosis of *Streptococcus pneumoniae*. *Infect. Immun.* 67 2327–2333. 10.1128/iai.67.5.2327-2333.1999 10225891 PMC115974

[B22] Knupp-Pereira P. A. Marques N. T. C. Teixeira L. M. Póvoa H. C. C. Neves F. P. G. (2019). Prevalence of PspA families and pilus islets among *Streptococcus pneumoniae* colonizing children before and after universal use of pneumococcal conjugate vaccines in Brazil. *Braz. J. Microbiol*. 51 419–425. 10.1007/s42770-019-00179-y 31802411 PMC7203294

[B23] Li J. Li J. W. Feng Z. Wang J. An H. Liu Y. (2016). Epigenetic switch driven by DNA inversions dictates phase variation in *Streptococcus pneumoniae*. *PLoS Pathog.* 12:e1005762 10.1371/journal.pone.1005762 PMC4948785 27427949

[B24] Li J. Zhang J. R. (2019). Phase variation of *Streptococcus pneumoniae*. *Microbiol. Spectr.* 7:e005-18. 10.1128/microbiolspec.GPP3-0005-2018 30737916 PMC11590436

[B25] Manso A. S. Chai M. H. Atack J. M. Furi L. De Ste Croix M. Haigh R. (2014). A random six-phase switch regulates pneumococcal virulence via global epigenetic changes. *Nat. Commun.* 5 1–9. 10.1038/ncomms6055 PMC4190663 25268848

[B26] Martin D. P. Murrell B. Golden M. Khoosal A. Muhire B. (2015). RDP4: detection and analysis of recombination patterns in virus genomes. *Virus Evol.* 1 1–5. 27774277 10.1093/ve/vev003 PMC5014473

[B27] McDaniel L. S. McDaniel D. O. Hollingshead S. K. Briles D. E. (1998). Comparison of the PspA sequence from *Streptococcus pneumoniae* EF5668 to the previously identified PspA sequence from strain Rx1 and ability of PspA from EF5668 to elicit protection against pneumococci of different capsular types. *Infect. Immun.* 66 4748–4754. 10.1128/iai.66.10.4748-4754.1998 9746574 PMC108585

[B28] Melin M. M. Hollingshead S. K. Briles D. E. Lahdenkari M. I. Kilpi T. M. Käyhty H. M. (2008). Development of antibodies to PspA families 1 and 2 in children after exposure to *Streptococcus pneumoniae*. *Clin. Vaccine Immunol.* 15 1529–1535. 10.1128/cvi.00181-08 18753341 PMC2565922

[B29] Metcalf B. J. Gertz R. E. Jr. Gladstone R. A. Walker H. Sherwood L. K. Jackson D. (2016). Strain features and distributions in pneumococci from children with invasive disease before and after 13-valent conjugate vaccine implementation in the USA. *Clin. Microbiol. Infect.* 22 e9–e29. 26363404 10.1016/j.cmi.2015.08.027 PMC4721534

[B30] Moschioni M. Donati C. Muzzi A. Masignani V. Censini S. Hanage W. P. (2008). *Streptococcus pneumoniae* contains 3 rlrA pilus variants that are clonally related. *J. Infect. Dis.* 197 888–896. 18269316 10.1086/528375

[B31] Nelson A. L. Ries J. Bagnoli F. Dahlberg S. Fälker S. Rounioja S. (2007). RrgA is a pilus-associated adhesin in *Streptococcus pneumoniae*. *Mol. Microbiol.* 66 329–340. 10.1111/j.1365-2958.2007.05908.x 17850254 PMC2170534

[B32] Ochs M. M. Bartlett W. Briles D. E. Hicks B. Jurkuvenas A. Lau P. (2008). Vaccine-induced human antibodies to PspA augment complement C3 deposition on *Streptococcus pneumoniae*. *Microb. Pathog.* 44 204–214. 10.1016/j.micpath.2007.09.007 18006268 PMC2288783

[B33] Pimenta F. C. Ribeiro-Dias F. Brandileone M. C. Miyaji E. N. Leite L. C. Sgambatti de Andrade A. L. (2006). Genetic diversity of PspA types among nasopharyngeal isolates collected during an ongoing surveillance study of children in Brazil. *J. Clin. Microbiol.* 44 2838–2843. 10.1128/jcm.00156-06 16891500 PMC1594641

[B34] Pinto T. C. Kegele F. C. Dias C. A. Barros R. R. Peralta J. M. Merquior V. L. (2016). *Streptococcus pneumoniae* serotypes 9 and 14 circulating in Brazil over a 23-year period prior to introduction of the 10-valent pneumococcal conjugate vaccine: role of international clones in the evolution of antimicrobial resistance and description of a novel genotype. *Antimicrob. Agents Chemother.* 60 6664–6672. 10.1128/aac.00673-16 27572394 PMC5075071

[B35] Pinto T. C. Souza A. R. de Pina S. E. Costa N. S. Borges Neto A. A. Neves F. P. (2013). Phenotypic and molecular characterization of optochin-resistant *Streptococcus pneumoniae* isolates from Brazil, with description of five novel mutations in the ATPC gene. *J. Clin. Microbiol.* 51 3242–3249. 10.1128/jcm.01168-13 23884994 PMC3811620

[B36] Regev-Yochay G. Hanage W. P. Trzcinski K. Rifas-Shiman S. L. Lee G. Bessolo A. (2010). Re-emergence of the type 1 pilus among *Streptococcus pneumoniae* isolates in Massachusetts, USA. *Vaccine* 28 4842–4846. 10.1016/j.vaccine.2010.04.042 20434550 PMC2897942

[B37] Rolo D. Ardanuy C. Fleites A. Martín R. Liñares J. (2009). Diversity of pneumococcal surface protein A (PspA) among prevalent clones in Spain. *BMC Microbiol.* 9:80. 10.1186/1471-2180-9-80 19419534 PMC2684541

[B38] Sadowy E. Kuch A. Gniadkowski M. Hryniewicz W. (2010). Expansion and evolution of the *Streptococcus pneumoniae* Spain9V-ST156 clonal complex in Poland. *Antimicrob. Agents Chemother.* 54 1720–1727. 10.1128/aac.01340-09 20194703 PMC2863602

[B39] Saluja S. K. Weiser J. N. (1995). The genetic basis of colony opacity in *Streptococcus pneumoniae*: evidence for the effect of box elements on the frequency of phenotypic variation. *Mol. Microbiol.* 16 215–227. 10.1111/j.1365-2958.1995.tb02294.x 7565084

[B40] Selva L. Ciruela P. Blanchette K. del Amo E. Pallares R. Orihuela C. J. (2012). Prevalence and clonal distribution of pcpA, psrP and Pilus-1 among pediatric isolates of *Streptococcus pneumoniae*. *PLoS One* 7:e41587. 10.1371/journal.pone.0041587 22848535 PMC3404996

[B41] Sjöström K. Blomberg C. Fernebro J. Dagerhamn J. Morfeldt E. Barocchi M. A. (2007). Clonal success of piliated penicillin nonsusceptible pneumococci. *Proc. Natl. Acad. Sci. U.S.A.* 104 12907–12912. 10.1073/pnas.0705589104 17644611 PMC1929012

[B42] Swiatlo E. Brooks-Walter A. Briles D. E. McDaniel L. S. (1997). Oligonucleotides identify conserved and variable regions of pspA and pspA-like sequences of *Streptococcus pneumoniae*. *Gene* 188 279–284. 10.1016/s0378-1119(96)00823-2 9133603

[B43] Vela-Coral M. C. Fonseca N. Castañeda E. Di Fabio J. L. Hollingshead S. K. Briles D. E. (2001). Pneumococcal surface protein A of invasive *Streptococcus pneumoniae* isolates from Colombian children. *Emerg. Infect. Dis.* 7, 832–836. 10.3201/eid0705.017510 11747695 PMC2631885

[B44] Weiser J. N. Austrian R. Sreenivasan P. K. Masure H. R. (1994). Phase variation in pneumococcal opacity: relationship between colonial morphology and nasopharyngeal colonization. *Infect. Immun.* 62 2582–2589. 10.1128/iai.62.6.2582-2589.1994 8188381 PMC186548

[B45] Weiser J. N. Ferreira D. M. Paton J. C. (2018). *Streptococcus pneumoniae*: transmission, colonization and invasion. *Nat. Rev. Microbiol.* 16 355–367. 10.1038/s41579-018-0001-8 29599457 PMC5949087

